# Histone methyltransferase KMT2D contributes to the protection of myocardial ischemic injury

**DOI:** 10.3389/fcell.2022.946484

**Published:** 2022-07-22

**Authors:** Shu-Bao Liu, Xiang-Min Meng, Yu-Meng Li, Jun-Meng Wang, Hui-Hui Guo, Chaochen Wang, Bing-Mei Zhu

**Affiliations:** ^1^ Regenerative Medicine Research Center, West China Hospital, Sichuan University, Chengdu, Sichuan, China; ^2^ Key Laboratory of Acupuncture and Medicine Research of Ministry of Education, Nanjing University of Chinese Medicine, Nanjing, Jiangsu, China; ^3^ Zhejiang University-University of Edinburgh Institute, International Campus, Zhejiang University, Haining, Zhejiang, China

**Keywords:** methyltransferase, serum, transcriptional regulation, glucocorticoid response element, ischemia

## Abstract

Histone H3 lysine 4 (H3K4) methyltransferase 2D (KMT2D) plays an important role in cell development in early life. However, the function of KMT2D in adult cells such as cardiomyocytes or neurons has not been reported. In this study, cardiomyocyte-specific KMT2D knockout (KMT2D-cKO) and control (KMT2D-Ctl) mice were exposed to sham or myocardial ischemia (MI) surgery. Depletion of KMT2D aggravated the ischemic area, led to the increased mortality (26.5% in KMT2D-cKO vs 12.5% in KMT2D-Ctl) of the mice, and weakened the left ventricular systolic function. RNA-seq analysis in cardiac tissues identified genes whose expression was changed by MI and KMT2D deletion. Combined with the genome-wide association study (GWAS) analysis, cardiac disease-associated genes *Rasd1*, *Thsd7a*, *Ednra*, and *Tns1* were identified. The expression of the *Rasd1* was significantly decreased by MI or the loss of KMT2D *in vivo*. Meanwhile, ChIP assays demonstrated that either MI or loss of KMT2D attenuated monomethylated H3K4 (H3K4me1) enrichment on the enhancer of *Rasd1*. By generating a KMT2D knockout (H9C2-KO) H9C2 monoclone, we verified that the expression of *Rasd1* was controlled by KMT2D, and the expression of *Rasd1* was decreased by serum starvation but not low-(O_2_) treatment in H9C2 cells. KMT2D has a protective effect on ischemic myocardium by regulating cardiac disease-associated genes including *Rasd1*. KMT2D is required for the H3K4me1 deposition on the enhancer of *Rasd1*. Our data for the first time suggest that KMT2D-mediated *Rasd1* expression may play an important protective effect on adult cells during nutritional deficiency caused by ischemic injury.

## Introduction

Histone H3 lysine 4 (H3K4) methyltransferase 2D (KMT2D) plays an important role in cell development in early life. However, it is not clear if they can affect the adult cells, such as cardiomyocytes or neurons, which have been shown lack the ability to regenerate (Blackshaw, 2022; Li et al., 2018). However, recent studies have shown that the neurons and cardiomyocytes can be improved in some way under the disease condition. After the onset of ischemia, epigenetic modifications of histones in cardiomyocytes are altered accordingly (K. Wang K et al., 2021). Recent studies have demonstrated that histone modifications, including methylation and acetylation, regulate gene transcription and exhibit crucial effects on metabolism, stress-resistance, survival, and apoptosis of adult cells (Li et al., 2020; Yang et al., 2017; Zhang et al., 2018). Methylation and acetylation of histones are dynamically catalyzed by methyltransferases/demethylases and acetylases/deacetylases, respectively (Hyun et al., 2017). Histone H3 lysine 4 (H3K4) methyltransferase KMT2D, also known as MLL2 in humans or MLL4 in mice, co-localizes with various transcription factors on the enhancers, and is responsible for the mono-methylation of H3K4 (H3K4me1) and transcriptional activation of target genes (Froimchuk et al., 2017; L. H. Wang L H et al., 2021). *Kmt2d* is frequently mutated in Kabuki syndrome (Bogershausen and Wollnik, 2013), about 70% of these patients are diagnosed with congenital heart diseases, or neuro-intellectual disability (Digilio et al., 2017; Van Laarhoven et al., 2015). Deactivation of *Kmt2d* in mice results in embryonic lethality at embryonic day 9.5–10.5 (Ang et al., 2016; Lee et al., 2013). Inactivation of *Kmt2d* in zebrafish demonstrated that *Kmt2d* is essential for heart development through activating notch signaling pathway (Serrano et al., 2019). *Kmt2d* knockout by *Mesp1*-Cre, which expresses in the precursors of cardiovascular system (Kitajima et al., 2006), results in severely cardiac defects and embryonic death in mice (Ang et al., 2016). Similarly, no alive offspring are produced while *Kmt2d* is conditionally knocked out by *Mef2c*AHF-Cre or *Tnnt2*-Cre, which is specifically expressed in anterior heart field (AHF) precursors or the embryonic myocardium and mature cardiomyocytes in adult heart, respectively (Ang et al., 2016). These results indicate that *Kmt2d* plays crucial roles in cell development, notably during cardiac organogenesis in the embryonic stage. However, it remains unclear what function KMT2D exerts in the adult cells, especially when cardiovascular disease occurs, such as ischemia.

To investigate the function of KMT2D in adult cells, we generated inducible *Kmt2d* knockout mice, and observed deteriorative myocardial ischemic injury. Further RNA-seq and clinical CAD genome-wide association studies (GWAS) (Erdmann et al., 2018) together with ChIP analysis identified a potential target gene, called Ras related dexamethasone-induced 1 (*Rasd1*), in adult cells exposed to ischemic stress.

*Rasd1*, also known as *Dexras1*, is a member of the Ras superfamily and is induced by dexamethasone and originally described as a small GTPase (Kemppainen and Behrend, 1998). The inactivation of *Rasd1* caused by epigenetic DNA methylation is closely associated with dexamethasone resistance in multiple myeloma (Nojima et al., 2009). Dexamethasone could significantly reduce ischemia/reperfusion damage and improves cardiac recovery after ischemia (Xue et al., 2014). Dexamethasone has been reported to reduce atherosclerosis, and protect the myocardium from acute ischemic injury in animal models (Libby et al., 1973; Ng and Celermajer, 2004). Clinical application of dexamethasone alongside statins could attenuate myocardial damage in patients with acute myocardial infarction (Alisky, 2006). Reeve et al., discovered that dexamethasone could reduce stress-induced apoptosis in the H9C2 cells (Reeve et al., 2007). Glucocorticoid receptor (GR) mediates dexamethasone induced *Rasd1* expression in islets (Lellis-Santos et al., 2012). Activation of GR by dexamethasone could prevent cardiac injury during myocardial ischemia (Xu et al., 2011). KMT2D interacts with GR to co-occupy glucocorticoid response elements (GREs) of the Sodium Channel Epithelial 1 Subunit Alpha (*Enaca*) and modulates its transcription in human retinal pigment epithelial cells following dexamethasone stimulation (Yang et al., 2020).

In heart disease, RASD1 was found to significantly inhibit the secretion of atrial natriuretic peptide (ANP) in HL-1 cells (McGrath et al., 2012). In this study, we designed both *in vivo* and *in vitro* experiments to exam the important role of *Kmt2d* and its relation with *Rasd1* in the stress condition. Our study revealed that KMT2D controls *Rasd1* expression and protects adult growth against ischemia possibly associated with *Rasd1*.

## Materials and methods

### Mice

*Kmt2d* ^*flox/flox*^ mice (Lee et al., 2013) were obtained from Dr. Kai Ge (National Institute of Diabetes and Digestive and Kidney Diseases, NIH, United States). *Tnnt2*-rtTA/tetO-Cre (cardiomyocyte-specific Cre) mice (Wu et al., 2010) were kindly donated by Dr. Bin Zhou (Shanghai Institutes for Biological Sciences of the Chinese Academy of Sciences, China). Female *Kmt2d* ^*flox/flox*^ mice were crossed with *Tnnt2*-Cre male mice and bred in the approved Experimental Animal Center at West China Hospital of Sichuan University (Chengdu, China) to generate the *Kmt2d* ^*flox/flox*^; *Tnnt2*-rtTA/tetO-Cre mice. The mice were housed in specific pathogen-free conditions (3-5 mice/cage, temperature at 20–26°C, relative humidity at 40–70% and 12 h dark/light cycle) and given free access to sterile feed and water. Genotyping procedure was performed by polymerase chain reaction (PCR) using genomic DNA. The sequences of relative primers are listed in Supplementary Table S1.

Eight-week-old *Kmt2d* ^*flox/flox*^; *Tnnt2*-rtTA/tetO-Cre male mice were fed with doxycycline (Dox) at a concentration of 2 mg/ml (Chen et al., 2018) in drinking water for 14 days to knockout *Kmt2d* in cardiomyocytes (KMT2D-cKO). One week after Dox withdrawal, experiments were carried out. Their *Kmt2d* ^*flox/flox*^ without *Tnnt2*-Cre littermates were used as control (KMT2D-Ctl).

All animal experimental protocols were reviewed and approved by the Institutional Animal Care and Use Committees of West China Hospital of Sichuan University (No. 2020267A).

### Ischemia mouse modeling

KMT2D-Ctl and KMT2D-cKO mice were anaesthetized with isoflurane, and fixed in a supine position. The neck and chest of mice were cleaned and disinfected with 75% absolute ethyl alcohol. The jugular anterior fascicles were bluntly dissected along the midline to expose the trachea through a central incision in the neck. The mice were intubated in trachea and the ventilator was used to assist breathing. An incision was created on left side of the sternum and the thorax was opened between the third and fourth costal cartilages. The heart was exposed, then the left anterior descending coronary artery was visualized using stereoscopic microscope and ligated with 7–0 silk suture. Schematic diagram visualized ligation of left anterior descending coronary artery for artificial myocardial ischemia (MI) mice model (Supplementary Figure S1A). Successful myocardial ischemia modeling was confirmed by epicardial cyanosis and raised ST-segment of the electrocardiogram (Supplementary Figures S1B,C). The chest and skin were then closed with 6–0 silk suture.

Mice were divided into four groups: control mice sham operation (KMT2D-Ctl-sham), control mice myocardial ischemia (KMT2D-Ctl-MI), KMT2D-cKO mice sham operation (KMT2D-cKO-sham), and KMT2D-cKO mice myocardial ischemia (KMT2D-cKO-MI). In this study, the 24-h time point of myocardial ischemia was selected. Myocardial tissues were harvested in the end of ischemia and the ischemic size was evaluated by TTC staining.

### Triphenyl tetrazolium chloride staining

The excised hearts were immediately transferred to pre-cooled (4°C) phosphate buffered saline (PBS) solution to rinse blood, and frozen at −20°C for 30 min. Then each frozen heart was cut transversely into five 2-mm-thick slices. The slices were immersed in 1% TTC (Sigma-Aldrich, United States) in PBS solution, and incubated at 37°C for 15 min followed by treatment of 4% (v/v) paraformaldehyde at 4°C for 12 h. The infarcted tissues were unstained and appeared pale, while viable tissues showed as red (Guo et al., 2021). The infarcted size (IS) and total left ventricular (LV) area were measured by Image-Pro Plus 6.0 software. The infarct size percentage was quantified for each slide by using the following formulas: IS (%) = IS/total LV area × 100.

### Immunofluorescent double staining

The hearts were harvested, washed with PBS, and fixed in 4% paraformaldehyde (Biosharp, Cat No. BL539A) at 4°C for 24 h, followed by incubation in 30% sucrose and then embedded in OCT at −80°C. Frozen sections (8-μm thickness) were processed and permeabilized using 0.3% Triton X-100 (Solarbio, Cat No. T8200) for 30 min, and then blocked with 1% bovine serum albumin (BioFroxx, Cat No. 4240GR) in PBS at 37°C for 1 h. The sections were incubated with primary antibodies: anti-TNNT2 (1:100, Abcam, Cat No. ab8295), anti-KMT2D (1:100, Merckmillipore, Cat No. ABE 1867), secondary antibody (1:500, Alexa Fluor 488, Proteintech, Cat No. SA00013-2; Alexa Fluor 568, Invitrogen, Cat No. A11031). The nuclear staining was performed with DAPI (Beyotime, Cat No. C1005). Immunofluorescences images were visualized using an OLYMPUS microscope BX43.

### RNA isolation

Heart tissue was ground into powder in liquid nitrogen and total RNA was isolated using TRIzol™ Reagent (Invitrogen™, Cat No. 15596026) according to the instructions. RNA purity and concentrations were confirmed by spectrophotometry (absorbance at 260/280nm; Thermo Scientific™ NanoDrop™ One).

### Quantitative real-time PCR

Reverse transcription of total RNA was performed using the HiScript II Q RT SuperMix (Vazyme, Cat No. R223-01). The qPCR was performed by CFX96™ Real-Time System (Bio-Rad Laboratories, United States), according to the instructions of TB Green^®^ Premix (Takara, Cat No. RR820A). The quantification of target gene in expression was calculated using the 2^−ΔΔCt^ method, and beta-Actin was used as a normalized reference. The primers were shown in Supplementary Table S2.

### Echocardiography

Heart function was determined 7 days after MI using a small animal high-resolution ultrasound (VIVID7, GE) equipped with a i13L imaging transducer. Briefly, mice were anesthetized with 1–2% isoflurane and chest hairs were removed. The left ventricular end-diastolic diameter (LVEDD) and left ventricular end-systolic diameter (LVESD) were recorded and measured in M-mode. The left ventricular ejection fraction (EF) and fractional shortening (FS) were calculated according to the formula [(LVEDD)^3^ − (LVESD)^3^]/(LVEDD)^3^ × 100% and (LVEDD − LVESD)/LVEDD × 100%, respectively.

### RNA-seq and bioinformatic analysis

The total RNA was extracted from the heart. After quality quantification, RNA sequencing was conducted by Shanghai Liebing Biomedical Technology Co., Ltd. The FPKM (fragments per kilobase of exon model per million mapped fragments) data with a large deviation from the mean and the FPKM with zero values were discarded. The remaining FPKM data were used for describing advanced volcano plot, venn diagram and heatmap of gene expression.

### GWAS data

The genes at genome-wide significant CAD risk loci were obtained from previous study (Erdmann et al., 2018). The orthologs in mice were identified by Gene ID Conversion Tool from DAVID (Huang et al., 2009a; Huang et al., 2009b).

### Western blotting

The cardiac samples were ground in liquid nitrogen and lysed with RIPA buffer (Beyotime, Cat No. P0013) with protease inhibitor cocktail (Roche, Cat No. 04693116001). Equivalent amounts of protein were separated by sodium dodecyl sulphate-polyacrylamide gel electrophoresis (SDS-PAGE), and transferred onto PVDF membranes (Merck Millipore, Cat No. ISEQ00010). Nonspecific reactivity was blocked in 5% skim milk in PBST (0.01M phosphate buffer solution, pH 7.2, 0.6% Tween-20) for 1 h at room temperature. The membranes were incubated with the specific primary antibodies against KMT2D (1:1000, Merck Millipore, Cat No. ABE 1867), RASD1 (1:800, Abcam, Cat No. ab251924) and β-Actin (1:1000, ZSGB-BIO, Cat No. TA-09) overnight at 4°C, then with the horseradish peroxidase (HRP)–conjugated secondary antibodies (1:3000, Invitrogen, Cat No. #31430 and #31460) for 3 h at room temperature. Signals were detected by the FUSION FX system (VILBER, France) using SuperSignal™ West Pico PLUS (Thermo, Cat No. 34580). The β-Actin was used as a normalized reference to assess protein levels.

### ChIP-qPCR assay

The 24 h post-sham/MI-operated tissues of hearts were harvested and ground into powder in liquid nitrogen. The powdered tissues were lysed and chromatin was extracted using the SimpleChIP^®^ Plus Sonication Chromatin IP Kit (Cell Signaling Technology, Cat No. #56383). The chromatin was sheared by sonication and genomic DNA was immunoprecipitated with a ChIP-grade anti-H3K4me1 antibody (Abcam, Cat No. ab8895) following the SimpleChIP^®^ Plus Sonication Chromatin IP Kit. The qPCR was performed by CFX96™ Real-Time System, according to the instructions of TB Green^®^ Premix (Takara, Cat No. RR820A). The quantification was calculated using the 2^−ΔΔCt^ method, and input DNA was used as a normalized reference. The primers were shown in Supplementary Table S3.

### Cell culture

H9C2 cells were obtained from Procell Life Science Technology Co., Ltd. (Wuhan, China) and cultured at 37 °C and 5% CO_2_ in DMEM with L-glutamine medium (Gibco, Cat No. C11995500BT), 1% penicillin–streptomycin (Gibco, Cat No. 15140-122), with 10% fetal bovine serum (FBS, Gibco, Cat No. 16140-071), unless noted otherwise, in 60 mm dishes (Thermo Scientific, Cat No. 150462), as previously described (Pesant et al., 2006) with some modifications. When the cells grew to 70% confluent, fresh culture medium was replaced absolutely. Twenty-4 hours later, cells were treated with 1% O_2_ 8 h for hypoxic studies or serum-free medium 2–3 h for serum starvation experiments. The control cells were concomitantly cultured in normal conditions.

### CRISPR/Cas9 plasmids construction and gene editing

The oligodeoxynucleotides of gRNAs were annealed and cloned into PX458 plasmids (Addgene, Cat No. #48138), named PX458-*Kmt2d*-gRNA1 and PX458-*Kmt2d*-gRNA2, respectively, and confirmed by sequencing. H9C2 cells were detached by 0.25% trypsin-EDTA solution (Gibco, Cat No. 25200-056) and transfected with CRISPR/Cas9 PX458-*Kmt2d* gRNA1/2 or empty control plasmids by Nucleofector™ X Kit (Lonza, Cat No. V4XC-2024) according to the manufacture’s protocol, then plated in DMEM with 10% FBS without pen/strep. Cells were cultured for 48 h at 37°C. The PX458 plasmid contained a green fluorescent protein (GFP) marker that can be used to screen GFP positive cells for H9C2-KO monoclonal H9C2 cell line by aseptic flow cytometry.

### Statistical analysis

GraphPad Prism 6.01 was used to analyze the data. The measurement data were presented as mean ± standard deviation. The student’s t-test was used for two-group comparison. One-way analysis of variance (ANOVA) was used for comparison between more than two groups, and Tukey’s post hoc test for pairwise comparison. Predictors were kept if they were significant at a *p* value <0.05.

## Results

### Loss of *Kmt2d* led to increased mortality of mice and aggravated ischemic injury

**FIGURE 1:**
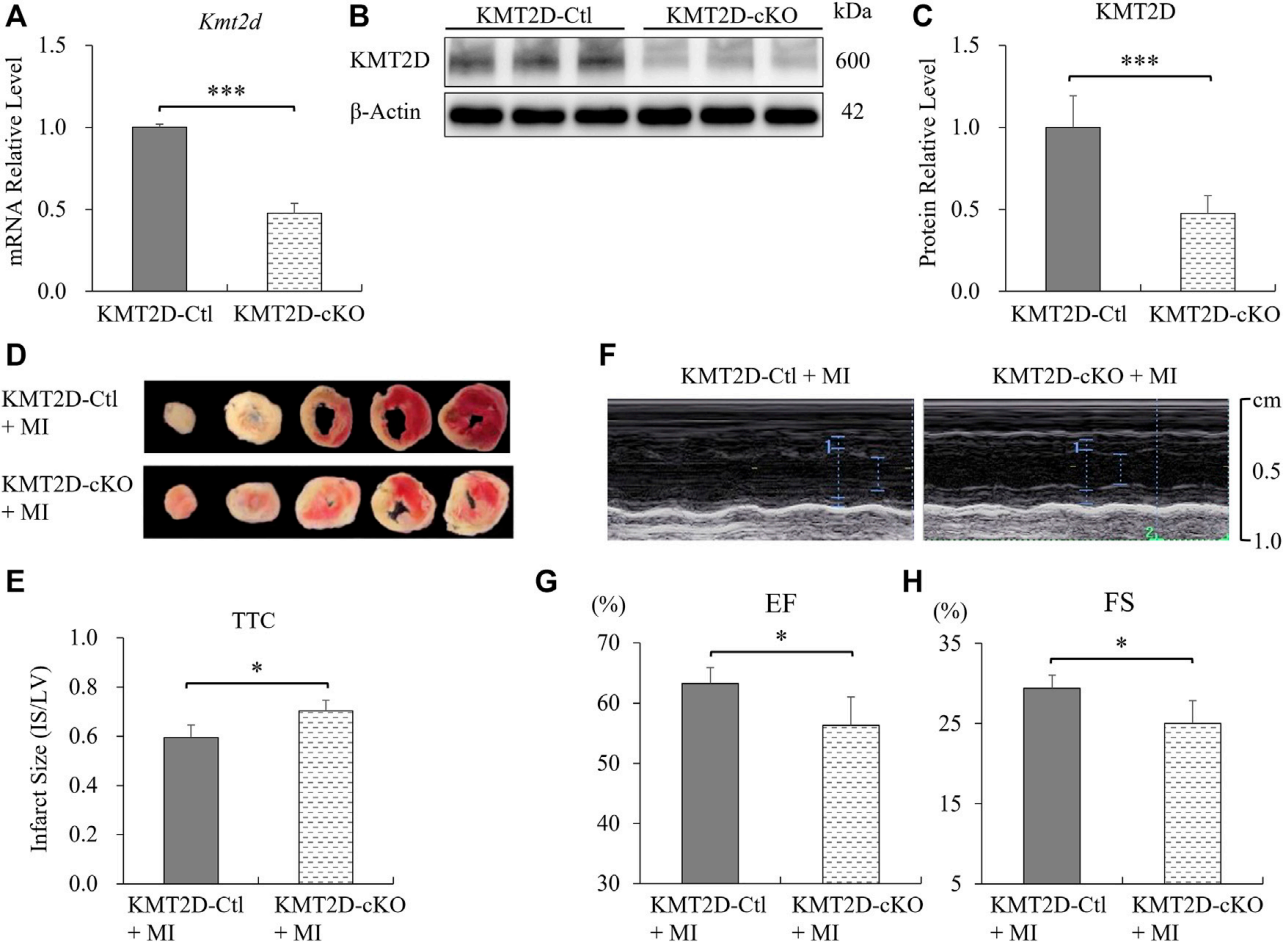
Knockout of *Kmt2d* aggravated ischemic injury. **(A)** Analysis of *Kmt2d* mRNA expression in the KMT2D-Ctl and KMT2D-cKO mice by qPCR. ***p < 0.001, *n* = 3. **(B)** Representative images of western blotting and **(C)** quantification analysis of KMT2D protein decrement of the KMT2D-cKO mice *vs*. KMT2D-Ctl mice. ***p < 0.001, *n* = 3. **(D)** Representative pictures of the TTC staining of KMT2D-cKO and KMT2D-Ctl mice 24 h post-MI. **(E)** Quantification of infarct size in the left ventricle by TTC staining. *p < 0.05, *n* = 5. **(F)** Representative images of echocardiography in two groups 7 days post-MI. **(G)** Left ventricular ejection fraction (EF) and **(H)** fractional shorting (FS) were analyzed. *p < 0.05, *n* = 4.

To evaluate the knockout efficiency of *Kmt2d*, adult myocardial tissues at the apex of the left ventricle were used to extract mRNAs and proteins. The qPCR and western blotting results showed that the products of *Kmt2d* gene were significantly reduced in the myocardium of KMT2D-cKO mice, to about 50% of those in the KMT2D-Ctl mice (Figures 1A–C). Immunofluorescence staining was used to confirm the specificity of KMT2D knockout in the cardiomyocytes. In the KMT2D-Ctl mice, KMT2D was co-expressed with TNNT2 in the heart tissues (Supplementary Figure S2A). However, In the KMT2D-cKO mice, KMT2D was almost undetectable in the TNNT2 positive cardiomyocytes (Supplementary Figure S2B). The mortality of myocardial ischemia within 24 h was calculated, and the result shows that the death rate in KMT2D-cKO mice was significantly higher (26.5%) than that in the KMT2D-Ctl group (12.5%), as shown in Table1.

**TABLE 1:** Mortality statistics of mice within 24 h of ischemia.

	KMT2D-Ctl	KMT2D-cKO	KMT2D-Ctl	KMT2D-cKO
	Sham	Sham	MI	MI
Total mice (Num.)	28	27	32	34
Survival (Num.)	26	26	28	25
Death (Num.)	2	1	4	9
Mortality (Num.)	7.1%	3.7%	12.5%	26.5%

Twenty-four hours after MI, adult hearts were harvested and the ischemic areas were analyzed by TTC staining. The ratio of infarct size to left ventricular (IS/LV) was calculated to evaluate the myocardial injury. The results show that the proportion of ischemic area in left ventricle in KMT2D-cKO mice was significantly higher than that in the KMT2D-Ctl group (Figures 1D,E). The left ventricular systolic function was assessed by echocardiography. The left ventricular ejection fraction (EF) and fractional shortening (FS) were significantly decreased in the KMT2D-cKO group compared to the KMT2D-Ctl mice 7 days post-MI (Figures 1F–H). These data indicate that specific knockout of *Kmt2d* in cardiomyocytes significantly increased the mouse mortality after MI, while the proportion of ischemic area in the left ventricle was increased as well, and the left ventricular systolic function was weakened, suggesting that *Kmt2d* is indispensable for the improved tolerance to cardiac ischemia in adult mice.

### *Kmt2d* deletion significantly affected gene transcription in adult tissue after ischemia.

**FIGURE 2:**
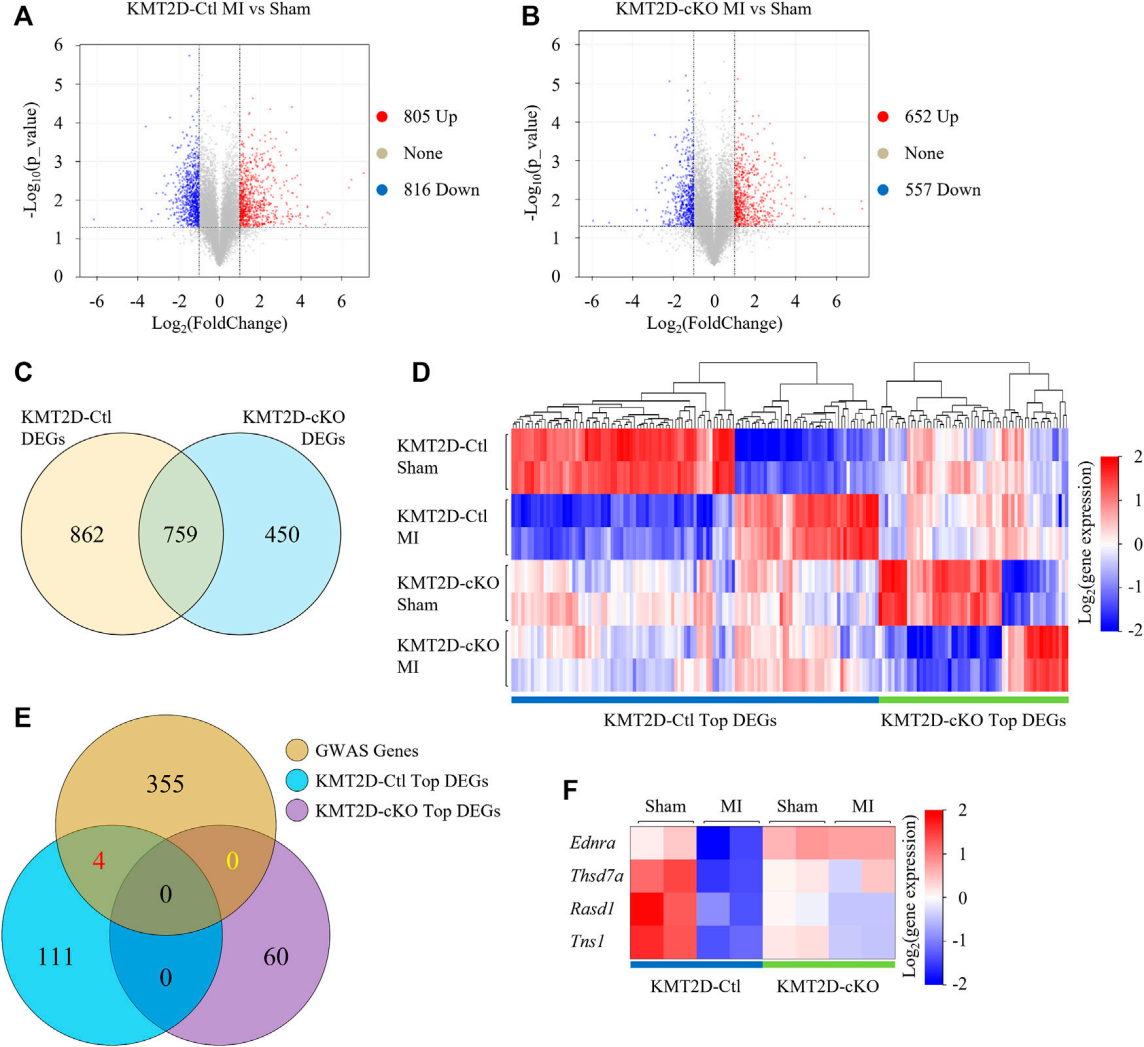
RNA-Seq analysis. **(A,B)** Volcano plot visualizes DEGs ( $\left|\log_{2}\left(FoldChange\right)\right|$ >1, *p* < 0.05, FDR<0.1) between MI and sham KO in the KMT2D-Ctl mice **(A)** and KMT2D-cKO mice **(B)**. Each point in the scatter plot represents a gene. Genes that up-regulated are shown in red and down-regulated in blue. Grey reflects genes whose expression levels are not significantly changed. **(C)** Venn diagram shows DEGs uniquely in KMT2D-Ctl mice hearts post-MI (862 genes), uniquely in the KMT2D-cKO mice hearts post-MI (450 genes), and in both (759 genes). **(D)** Heatmap is used to depict the top 115 DEGs after MI in the KMT2D-Ctl mice but no change in the KMT2D-cKO mice and the top 60 DEGs after MI in the KMT2D-cKO mice but no change in the KMT2D-Ctl mice. **(E)** Venn diagram displays the number of DEGs that associates with KMT2D during MI and genes from GWAS of human coronary artery disease. **(F)** Heatmap shows the transcription profile of the four DEGs in all groups.

To study the causes of aggravation of cell injury induced by KMT2D deletion after ischemia, we performed RNA-seq and determined transcriptional profiles in the sham and the 24-hours-post-MI hearts in the KMT2D-cKO and the KMT2D-Ctl mice. Transcriptomic analysis revealed 1,621 genes differentially expressed ( $\left|\log_{2}\left(FoldChange\right)\right|$ >1) in the MI group compared to the sham group of the KMT2D-Ctl mice, including 805 up-regulated and 816 down-regulated genes (Figure 2A; *p* < 0.05, FDR<0.1). The expression of 1,209 genes was significantly altered ( $\left|\log_{2}\left(FoldChange\right)\right|$ >1) by MI in the KMT2D-cKO mice, including 652 up-regulated and 557 down-regulated genes (Figure 2B; *p* < 0.05, FDR<0.1). Among the differential expressed genes (DEGs), 862 genes were specifically altered by MI in KMT2D-Ctl mice, while 450 genes were uniquely changed after MI in the KMT2D-cKO group (Figure 2C).

Then we focused on genes whose expression was altered by MI in the KMT2D-Ctl mice but not in the KMT2D-cKO mice, or vice versa. Top 115 of the 862 DEGs and top 60 of the 450 DEGs were identified, respectively (Figure 2D, Supplementary Table S4). To further screen out the truly important genes, we then examined if any of these genes was associated with coronary artery disease by analyzing the data from genome-wide associated studies (GWAS) (Erdmann et al., 2018). In the NCBI *Homo sapiens* database, 364 genes at the genomic significant CAD risk loci were identified with the gene IDs (Supplementary Table S5), and 359 of them have murine orthologs (Supplementary Table S6). Among these genes, venn analysis showed that four DEGs were in our list, namely *Rasd1* (Ras Related Dexamethasone Induced Protein 1), Thsd7a (Thrombospondin, type I, domain containing 7A), Ednra (Endothelin Receptor Type A), and Tns1 (Tensin 1) (Figure 2E). The expression of the *Rasd1* gene was significantly decreased by MI or the loss of KMT2D (Figure 2F).

### Expression of *Rasd1* was significantly reduced by acute ischemia and *Kmt2d* deletion in mice

**FIGURE 3:**
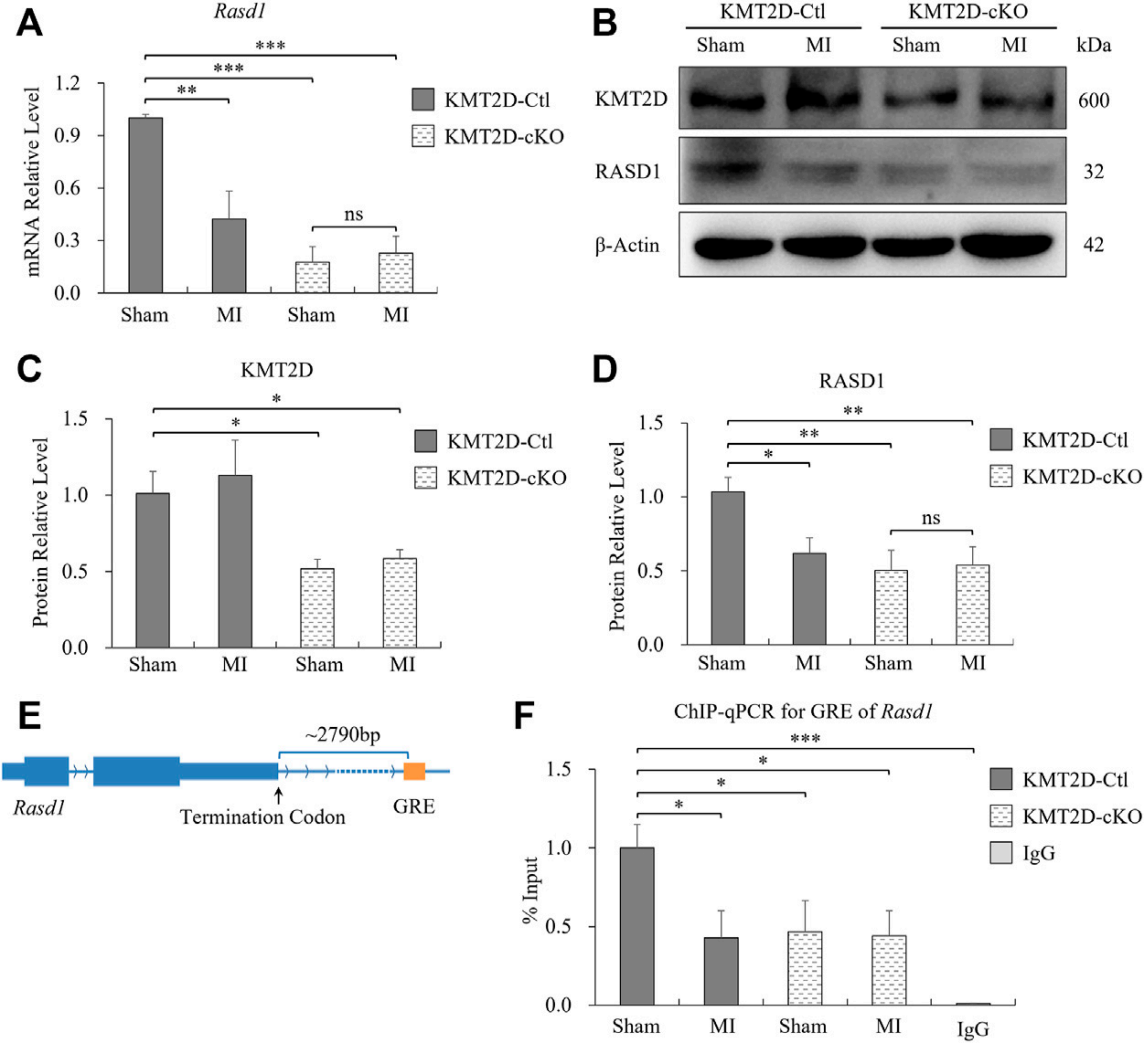
Expression of *Rasd1* in the heart was significantly reduced by ischemia and KMT2D deletion in mice. **(A)** qPCR measurements of *Rasd1* following 24 h post-MI or sham surgery in the KMT2D-Ctl and the KMT2D-cKO mice. **(B)** Representative images of western blots for KMT2D and RASD1, showing expression in MI/sham-operated hearts in the KMT2D-Ctl and the KMT2D-cKO mice. **(C)** Quantitative analysis of KMT2D expression in the KMT2D-cKO mice and the KMT2D-Ctl mice. **(D)** Quantitative analysis of RASD1 expression in the KMT2D-Ctl and the KMT2D-cKO mice during MI and sham groups. **(E)** Schematic diagram for the GRE motif of *Rasd1* gene. **(F)** ChIP-qPCR data quantifying enrichment of H3K4me1 at the GRE of *Rasd1* enhancer in all groups. (*n* = 3 of each group; *p < 0.05, **p < 0.01, ***p < 0.001.)

To further verify our findings, we collected ischemic heart tissues, including the infarct area and the border zone, from the 24-hours-MI and sham-operated group of the KMT2D-cKO and the KMT2D-Ctl mice. Down-regulated expression of *Rasd1* was detected in 24-hours-MI hearts compared with sham-operated ones in the KMT2D-Ctl mice at both mRNA (Figure 3A) and protein (Figures 3B,D) levels by qRT-PCR and western blot, respectively. Interestingly, the loss of KMT2D also led to a significant decrease in *Rasd1* expression in the heart (Figures 3A–D), consisting with the RNA-seq result. However, the transcription of *Rasd1* was not changed in the 24-hours-MI compared to the sham-operated hearts in the KMT2D-cKO mice (Figures 3A,D). The results suggested that KMT2D might be one of the upstream regulators of *Rasd1* gene.

### The H3K4me1 level was reduced on the GRE motif of *Rasd1* enhancer in *Kmt2d* knockout mice

KMT2D is one of the prominent H3K4 mono-methyltransferases shaping enhancers. Therefore, we speculated that the effect of KMT2D on *Rasd1* expression might be through H3K4 methylation regulation on the enhancer of *Rasd1*. Previous study identified a glucocorticoid response element (GRE) located about 2,300bp at the 3′-end of the *Rasd1* gene, which is a critical enhancer of *Rasd1* in human AtT-20 cells (Kemppainen et al., 2003). Interestingly, a GRE consensus element, GGTACATACTGTTCC was also located approximately 2,790bp downstream of the murine *Rasd1* (Figure 3E). Therefore, it was speculated that this sequence might be the core GRE fragment of *Rasd1* enhancer in mice. ChIP-qPCR results illustrated that, compared with sham-operated KMT2D-Ctl mice, H3K4me1 on the *Rasd1* GRE was significantly decreased in MI-operated KMT2D-Ctl mice, sham-operated KMT2D-cKO mice and MI-operated KMT2D-cKO mice (Figure 3F), suggesting that either *Kmt2d* deletion or MI in cardiomyocytes could lead to the reduction of H3K4me1 in GRE of the murine *Rasd1* enhancer, and further reduced *Rasd1* transcription in the heart tissue.

### Expression of *Rasd1* was weakened in the H9C2 cell line with *Kmt2d* deletion.

**FIGURE 4:**
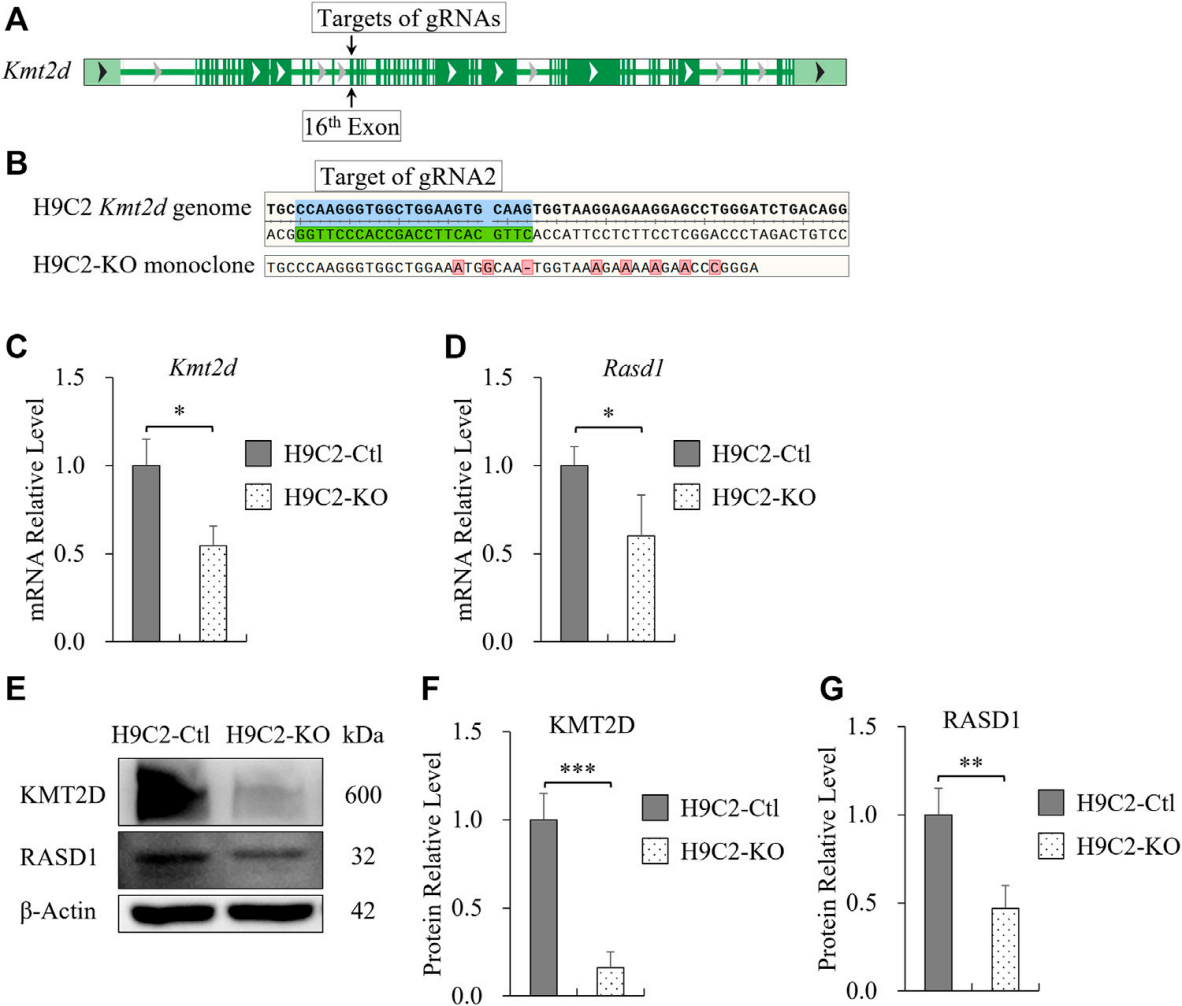
Transcription of *Rasd1* was weakened in the Monoclonal H9C2 cell line with deletion of KMT2D. **(A)** Schematic diagram showing targets of gRNAs on the 16th exon of *Kmt2d* gene in H9C2 cell. **(B)** Sanger sequencing of genomic DNA from CRISPR/Cas9-edited H9C2 monoclone. **(C,D)** qPCR analysis of *Kmt2d* **(C)** and *Rasd1* **(D)** expression in the H9C2-Ctl and the H9C2-KO monoclone. **(E)** Representative images of western blots for KMT2D and RASD1, showing expression in the H9C2-Ctl and the H9C2-KO cells. **(F)** Quantitative analysis of KMT2D expression in the H9C2-KO and the H9C2-Ctl monoclone. **(G)** Quantitative analysis of RASD1 expression in the H9C2-Ctl cell and the H9C2-KO monoclone. (*n* = 3 of each group; *p < 0.05, **p < 0.01, ***p < 0.001.)

To verify our findings and specifically investigate the effect of KMT2D on *Rasd1* expression in cell culture, *Kmt2d* gene was knocked out in the H9C2 cell line using CRISPR/Cas9 system *in vitro*. The sequences, spanning 16th-19th exons, were the knockout fragment of *Kmt2d* in our transgenic mouse model. Therefore, CRISPR guide RNAs (gRNAs) were designed to target the 16th exon (gRNA-1: 5′-GCT​TAA​GGG​CTG​GCG​TTG​TGt​gg-3′, gRNA-2: 5′-CTT​GCA​CTT​CCA​GCC​ACC​CTt​gg-3′) of *Kmt2d* in H9C2 (Figure 4A). After monoclonal selection, H9C2-KO cells were successfully established and confirmed by genomic sequencing (Figure 4B). The mRNA and protein of *Kmt2d* were significantly reduced in the H9C2-KO cells, compared with control H9C2 cells (Figures 4C–F). In line with our finding *in vivo*, expression of *Rasd1* was also weakened in H9C2-KO cells (Figures 4D,E,G). These results further confirmed that KMT2D regulates the expression of *Rasd1*.

### The decreased expression of *Rasd1* was not caused by hypoxia but by serum deficiency.

**FIGURE 5:**
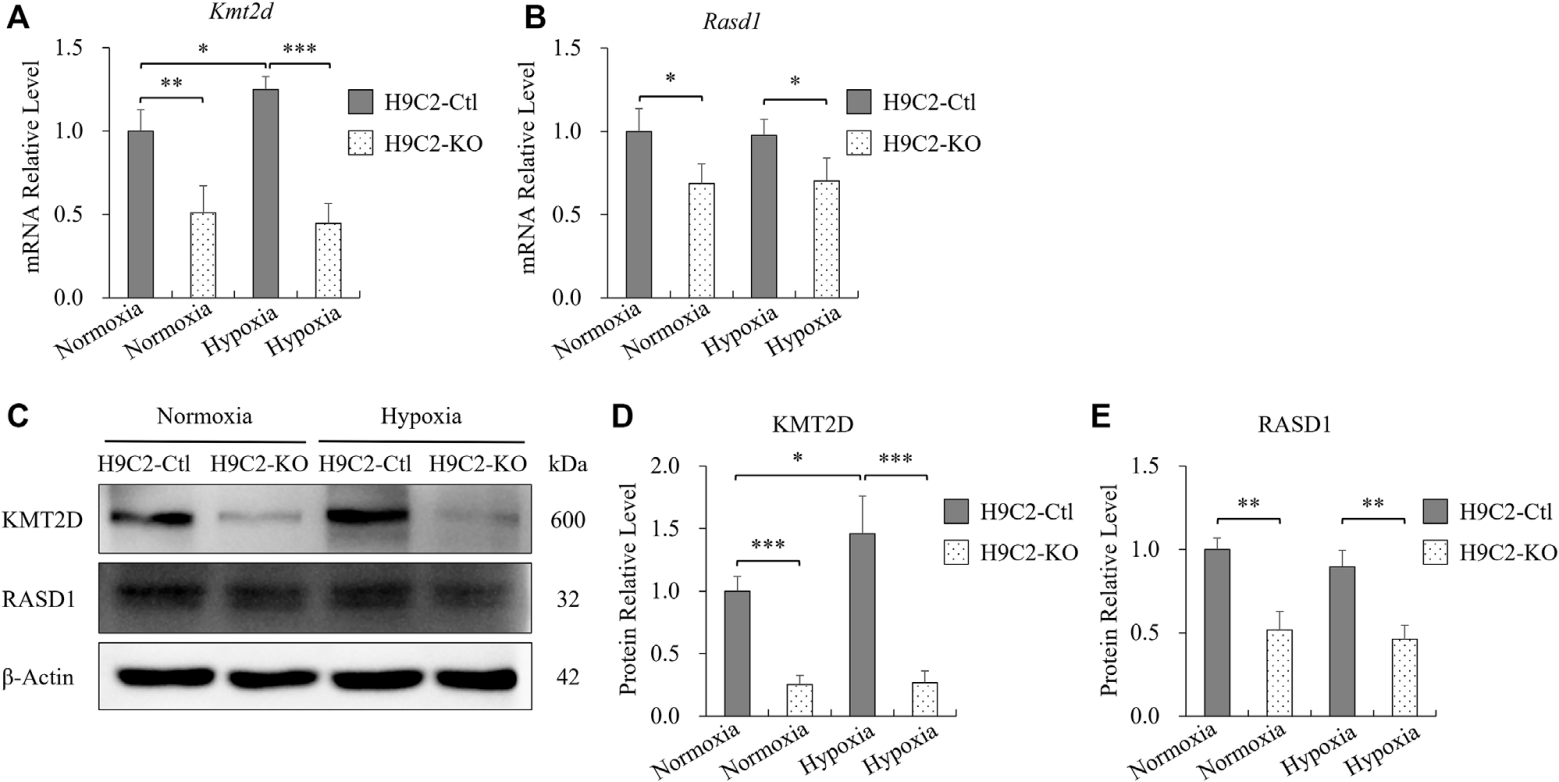
The expression of *Rasd1* in H9C2 cells was not affected by hypoxia. **(A,B)** qPCR analysis for *Kmt2d* **(A)** and *Rasd1* **(B)** in hypoxia in H9C2-Ctl and H9C2-KO cells. **(C)** Representative images of western blots for KMT2D and RASD1 expression in normoxia/hypoxia conditions in the H9C2-Ctl and H9C2-KO cells. **(D,E)** Quantitative analysis of KMT2D **(D)** and RASD1 **(E)** expression in the H9C2-Ctl or the H9C2-KO cells. (*n* = 3 of each group; *p < 0.05, **p < 0.01, ***p < 0.001.)

To further investigate whether the protective effect of KMT2D on MI was associated with RASD1, H9C2 cells were treated with 1% oxygen to simulate the hypoxic injury during MI. The *Kmt2d* expression in the control H9C2 was slightly increased in hypoxia (1% O_2_, 8 h) compared to the normoxia (Figures 5A,C,D). However, in the control H9C2, there was no significant differences of *Rasd1* expression between hypoxia and normoxia conditions (Figures 5B,C,E). In the H9C2-KO cell line, both mRNA and protein levels of *Kmt2d* and *Rasd1* were significantly decreased, compared with the control H9C2, but were also not affected by hypoxia (Figures 5B,C,E). These results suggested that down-regulation of *Rasd1* expression in ischemia might not be caused by hypoxia.

**FIGURE 6:**
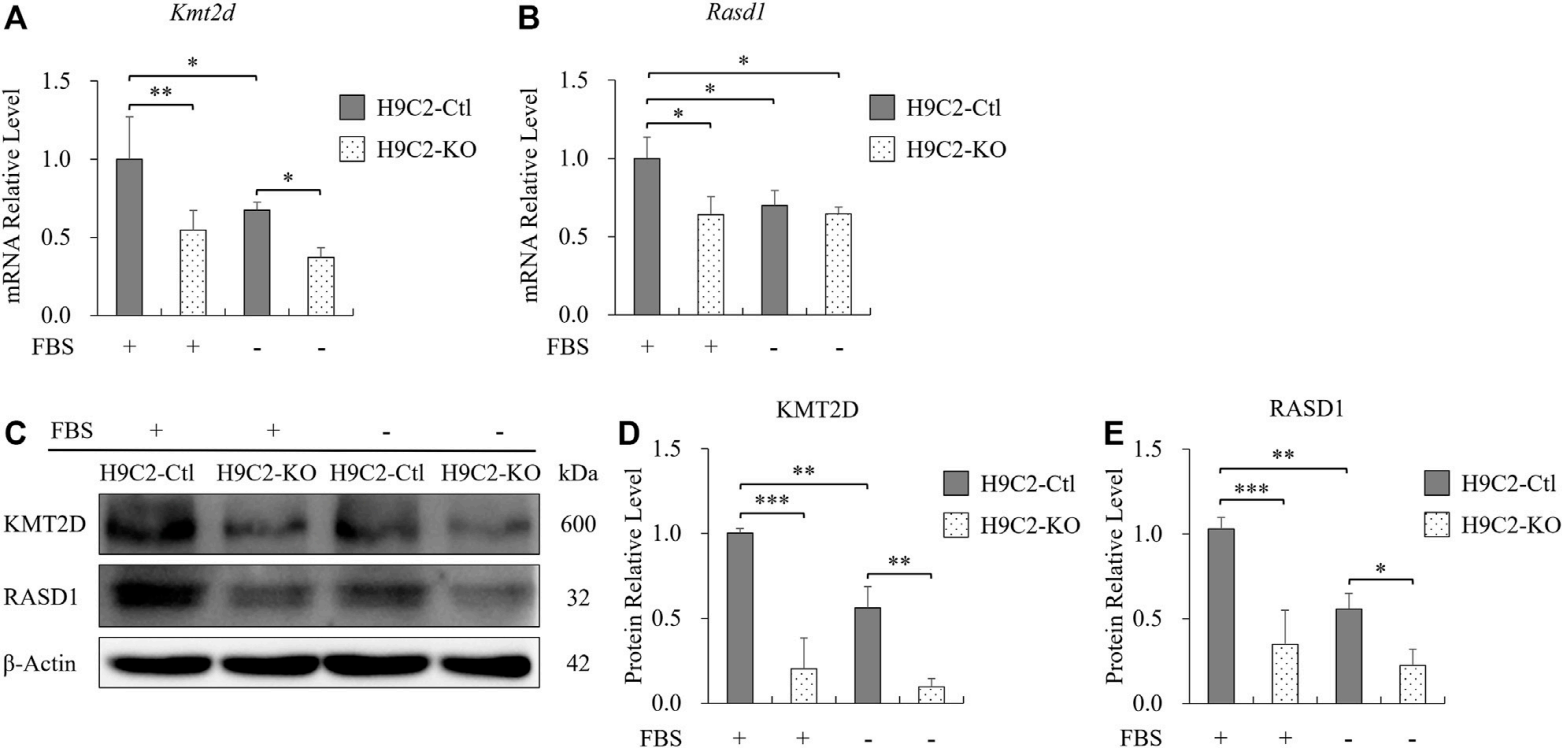
The decreased expression of *Rasd1* was caused by serum deficiency. **(A,B)** qPCR analysis for *Kmt2d* **(A)** and *Rasd1* **(B)** in FBS or FBS-free conditions in H9C2 cells. **(C)** Representative images of western blots for KMT2D and RASD1 in FBS or FBS-free conditions in the H9C2-Ctl and the H9C2-KO cells. **(D,E)** Quantitative analysis of KMT2D **(D)** and RASD1 **(E)** expression in the H9C2-Ctl and the H9C2-KO cell. (*n* = 3 of each group; *p < 0.05, **p < 0.01, ***p < 0.001.)

During ischemia, adult cells indeed were not only deprived of oxygen, but also deficient in nutrition from serum. Therefore, we then treated H9C2 cells with serum-free medium to mimic nutritional deficiency in myocardial ischemia. After 2 h of serum starvation, the mRNA level of *Kmt2d* was significantly reduced compared with the normal culture conditions in control H9C2 cells (Figure 6A), and a similar trend of *Rasd1* was also observed (Figure 6B). Three hours later of serum deprivation, the protein levels of KMT2D and RASD1 were both downregulated in the serum free condition from the H9C2-Ctl cells (Figures 6C–E). In the H9C2-KO cells, expressions of *Kmt2d* and *Rasd1* maintained at a low level within serum or not, and were significantly lower than those in the H9C2-Ctl group (Figures 6C–E). These data suggest that serum depletion downregulated both *Kmt2d* and *Rasd1* expression in adult cardiomyocytes, and that *Rasd1* was only sensitive to serum free but not to hypoxia.

## Discussion

During ischemia, adult tissues are under the stress of hypoxia and nutrient deficiency. In the initial stage of ischemia, gene transcription of adult cells, including cardiomyocytes and neurons changed dramatically to adapt to the ischemic environment. To avoid eventually developing into irreversible damage, the early therapeutic intervention might greatly improve the viability of cells. Therefore, detecting changes of functional genes in the early stage of ischemia and understanding how epigenetic modification could play an important role in regulating downstream genes is valuable for finding appropriate treatments to improve the resistance and survivability of cells in the heart. Thus, the current *in vivo* study was performed at 24 h post-MI.

In this study, we discovered that KMT2D exerted a protective effect on adult cells against MI. Deletion of *Kmt2d* led to increased injury and higher mortality of adult mouse during 24 h post-MI. As an H3K4 methyltransferase, KMT2D was widely expressed in many kinds of cells (Fagerberg et al., 2014) at multiple life stages, including adult cardiomyocytes. The protective effect of KMT2D on the ischemic was confirmed for the first time in this study.

Transcriptome analysis of KMT2D-Ctl and cKO mice uncovered changes of gene transcription in cardiac ischemic tissues after MI by the comparison with the sham-operated cardiac tissues. In this study, surgical ligation of the left anterior descending coronary artery was used to simulate the myocardial ischemia, which was very different from the process of clinical myocardial infarction. Therefore, we referred to the clinical GWAS data related to human coronary artery disease. Interestingly, *Rasd1* was found to be involved in the MI and might be a candidate down-stream gene of KMT2D.

*Rasd1* was induced by dexamethasone (Kemppainen and Behrend, 1998) which was commonly used as an anti-inflammatory glucocorticoid in various disorders including heart diseases (Dieleman et al., 2012; Maddali et al., 2019), autoimmune diseases, and allergic diseases (Iuchi et al., 2003). Dexamethasone suppressed expression of inflammatory genes NF-κB, IκBα, MMP-9, and TIMP-1 in patients with CAD (Jonsson et al., 2018). Previous study reported that dexamethasone treatment could retard the spread of the developing infarct within the ischemic myocardium (Lefer et al., 1980), and improve cardiac contractile performance in aged myocardium (Narayanan et al., 2004). But dexamethasone therapy was often limited by several adverse reactions due to long-term use or overdose (Iuchi et al., 2003; Ross and Linch, 1982). *Salmonella* enteritidis infection resulted in upregulation of interleukin-6 and some chemokines accompanied by the reduction of *Rasd1* in chicken granulosa cells (Tsai et al., 2010). It indicates that *Rasd1*, as a downstream gene, may participate in some anti-inflammatory effects of dexamethasone.

The expression of *Rasd1* was regulated by GR (Lellis-Santos et al., 2012). Early clinical studies had found that the density of GR decreased significantly in blood samples of patients with acute myocardial infarction within 24 h (Entzian et al., 1992). In our study, we discovered that the *Rasd1* expression was down-regulated 24 h post-MI in cardiac tissues of mice. In ARPE-19 human retinal pigment cells, KMT2D bound to GR, formed a complex, and participated in the regulation of GR-mediated expression of Sodium Channel Epithelial 1 Subunit Alpha (*Enaca*) (Yang et al., 2020). It was suggested that the reduction of *Rasd1* expression caused by KMT2D knockout may be related to GR in the heart, hinting that KMT2D may participate in the glucocorticoid-GR signaling pathway, which regulates inflammatory gene transcriptions.

Glucocorticoid response element (GRE) is an important regulatory region of *Rasd1* and a key binding site for GR in the enhancer (Kemppainen et al., 2003). H3K4me1 at enhancer regions catalyzed by KMT2D could activate the enhancers and promote gene transcription (Zheng et al., 2021). Interestingly, using ChIP qPCR assay, we found that H3K4me1 at GRE region of the *Rasd1* enhancer was weakened in MI or KMT2D-knockout cardiac tissues. This may lead to the reduction of transcriptional activity of *Rasd1* after MI or KMT2D deletion, which is consistent with the qPCR and western blotting results *in vivo*. Our current study suggested that the decrease of *Rasd1* at its endogenous level in the condition of myocardial ischemia may worsen myocardial ischemic injury. Therefore, *Rasd1* may be a potential anti-inflammatory therapeutic target, which could reduce the adverse effects of dexamethasone that is widely used clinically, especially when the patients have myocardial ischemia. Additional studies are required to further characterize the role of *Rasd1* in coronary heart diseases.

*In vitro*, we found that the expression of *Kmt2d* was slightly increased under hypoxia, compared to the normal conditions in H9C2 cells. Previous researches reported that KMT2D was involved in positively regulation of oxygen-responsive metabolic gene transcription in neuronal cells (Carosso et al., 2019), and genes related to hypoxia-reoxygenation in embryonic hearts (Ang et al., 2016). However, the response of *Kmt2d* expression to hypoxia had not been described until this study. Unexpectedly, *Rasd1* expression was not different between hypoxia and normoxia culture conditions in H9C2 cells, and its expression in H9C2-KO cells was not affected by hypoxia either. Moreover, we discovered that the loss of KMT2D in H9C2 cells induced *Rasd1* downregulation both at mRNA and protein levels. These results suggest that *Rasd1* might be insensitive to the hypoxic stimulation in H9C2 cells, and that the regulatory effect of KMT2D on *Rasd1* in H9C2 cells may be different from oxygen-responsive metabolic genes (Carosso et al., 2019).

During ischemia, adult cells suffered from not only hypoxia but also serum deficiency. Therefore, we cultured H9C2 cells in serum-free DMEM medium to mimic the state of nutrient loss after acute myocardial ischemia and to determine the expression of *Rasd1* and *Kmt2d* in the H9C2 cells. Interestingly, the expression of *Rasd1* was reduced after 3 h of serum deprivation in the H9C2, which was consistent with the downregulation of *Rasd1* after myocardial ischemia *in vivo*. Meanwhile, *Kmt2d* expression in H9C2 was also decreased in the serum-free DMEM medium. These data further emphasize that, to mimic myocardial ischemia *in vitro*, hypoxia is not enough, but more comprehensive factors, such as nutritional shortage should be considered as well.

## Conclusion

**FIGURE 7:**
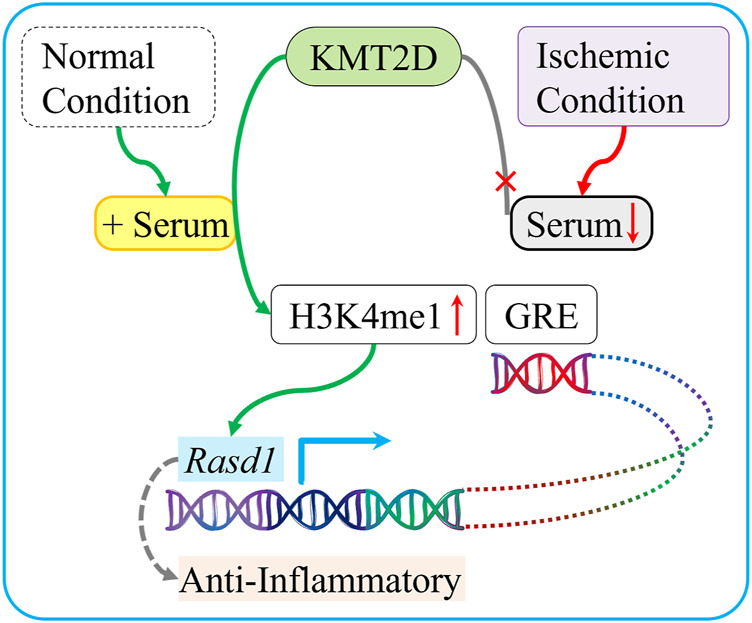
Schematic diagram summarizing the protective role of KMT2D during ischemia and the potential mediated mechanism. In normal condition, which is enriched with serum, KMT2D catalyzes H3K4 mono-methylation on the *Rasd1* enhancer region and promotes *Rasd1* transcription in cardiomyocyte. In ischemic condition, serum deficiency leads to interruption of H3K4me1 that modified by KMT2D on GRE of *Rasd1* and then reduces *Rasd1* transcription.

This study suggests that KMT2D directly methylates H3K4, which in turn binds to *Rasd1* GRE motifs in its enhancers, and promotes its transcription in the presence of serum. KMT2D exerts a protective effect against ischemia possibly related to the enhanced *Rasd1* transcription (Figure 7).

## Data Availability

The datasets presented in this study can be found in online repositories. The names of the repository/repositories and accession number(s) can be found below: https://www.ncbi.nlm.nih.gov/, PRJNA838783.

## References

[B1] Alisky J. M. (2006). Dexamethasone could improve myocardial infarction outcomes and provide new therapeutic options for non-interventional patients. Med. Hypotheses 67 (1), 53–56. 10.1016/j.mehy.2005.12.034 16503095

[B2] Ang S. Y. Uebersohn A. Spencer C. I. Huang Y. Lee J. E. Ge K. (2016). Kmt2d regulates specific programs in heart development via histone h3 lysine 4 di-methylation. Development 143 (5), 810–821. 10.1242/dev.132688 26932671 PMC4813342

[B3] Blackshaw S. (2022). Why has the ability to regenerate following cns injury been repeatedly lost over the course of evolution? Front. Neurosci. 16, 831062. 10.3389/fnins.2022.831062 35185460 PMC8854365

[B4] Bogershausen N. Wollnik B. (2013). Unmasking kabuki syndrome. Clin. Genet. 83 (3), 201–211. 10.1111/cge.12051 23131014

[B5] Carosso G. A. Boukas L. Augustin J. J. Nguyen H. N. Winer B. L. Cannon G. H. (2019). Precocious neuronal differentiation and disrupted oxygen responses in kabuki syndrome. JCI Insight 4 (20), 129375. 10.1172/jci.insight.129375 31465303 PMC6824316

[B6] Chen H. Jing X. Y. Shen Y. J. Wang T. L. Ou C. Lu S. F. (2018). Stat5-dependent cardioprotection in late remote ischaemia preconditioning. Cardiovasc. Res. 114 (5), 679–689. 10.1093/cvr/cvy014 29365089

[B7] Dieleman J. M. Nierich A. P. Rosseel P. M. van der Maaten J. M. Hofland J. Diephuis J. C. (2012). Intraoperative high-dose dexamethasone for cardiac surgery: A randomized controlled trial. JAMA 308 (17), 1761–1767. 10.1001/jama.2012.14144 23117776

[B8] Digilio M. C. Gnazzo M. Lepri F. Dentici M. L. Pisaneschi E. Baban A. (2017). Congenital heart defects in molecularly proven kabuki syndrome patients. Am. J. Med. Genet. A 173 (11), 2912–2922. 10.1002/ajmg.a.38417 28884922

[B9] Entzian P. Heer A. H. Leimenstoll G. Barth J. (1992). A microtitre assay system for glucocorticoid receptors: Decreased receptor concentration in myocardial infarction. Scand. J. Clin. Lab. Invest.. 52 (3), 169–175. 10.3109/00365519209088781 1411249

[B10] Erdmann J. Kessler T. Munoz V. L. Schunkert H. (2018). A decade of genome-wide association studies for coronary artery disease: The challenges ahead. Cardiovasc. Res. 114 (9), 1241–1257. 10.1093/cvr/cvy084 29617720

[B11] Fagerberg L. Hallstrom B. M. Oksvold P. Kampf C. Djureinovic D. Odeberg J. (2014). Analysis of the human tissue-specific expression by genome-wide integration of transcriptomics and antibody-based proteomics. Mol. Cell. Proteomics 13 (2), 397–406. 10.1074/mcp.M113.035600 24309898 PMC3916642

[B12] Froimchuk E. Jang Y. Ge K. (2017). Histone h3 lysine 4 methyltransferase kmt2d. Gene 627, 337–342. 10.1016/j.gene.2017.06.056 28669924 PMC5546304

[B13] Guo H. H. Jing X. Y. Chen H. Xu H. X. Zhu B. M. (2021). Stat3 but not stat5 contributes to the protective effect of electroacupuncture against myocardial ischemia/reperfusion injury in mice. Front. Med. 8, 649654. 10.3389/fmed.2021.649654 PMC8299366 34307396

[B14] Huang D. W. Sherman B. T. Lempicki R. A. (2009a). Bioinformatics enrichment tools: Paths toward the comprehensive functional analysis of large gene lists. Nucleic Acids Res. 37 (1), 1–13. 10.1093/nar/gkn923 19033363 PMC2615629

[B15] Huang D. W. Sherman B. T. Lempicki R. A. (2009b). Systematic and integrative analysis of large gene lists using david bioinformatics resources. Nat. Protoc. 4 (1), 44–57. 10.1038/nprot.2008.211 19131956

[B16] Hyun K. Jeon J. Park K. Kim J. (2017). Writing, erasing and reading histone lysine methylations. Exp. Mol. Med. 49 (4), e324. 10.1038/emm.2017.11 28450737 PMC6130214

[B17] Iuchi T. Akaike M. Mitsui T. Ohshima Y. Shintani Y. Azuma H. (2003). Glucocorticoid excess induces superoxide production in vascular endothelial cells and elicits vascular endothelial dysfunction. Circ. Res. 92 (1), 81–87. 10.1161/01.res.0000050588.35034.3c 12522124

[B18] Jonsson S. Lundberg A. K. Chung R. Jonasson L. (2018). Glucocorticoid sensitivity and inflammatory status of peripheral blood mononuclear cells in patients with coronary artery disease. Ann. Med. 50 (3), 260–268. 10.1080/07853890.2018.1445278 29473427

[B19] Kemppainen R. J. Behrend E. N. (1998). Dexamethasone rapidly induces a novel ras superfamily member-related gene in att-20 cells. J. Biol. Chem. 273 (6), 3129–3131. 10.1074/jbc.273.6.3129 9452419

[B20] Kemppainen R. J. Cox E. Behrend E. N. Brogan M. D. Ammons J. M. (2003). Identification of a glucocorticoid response element in the 3'-flanking region of the human dexras1 gene. Biochim. Biophys. Acta 1627 (2-3), 85–89. 10.1016/s0167-4781(03)00079-4 12818426

[B21] Kitajima S. Miyagawa-Tomita S. Inoue T. Kanno J. Saga Y. (2006). Mesp1-nonexpressing cells contribute to the ventricular cardiac conduction system. Dev. Dyn. 235 (2), 395–402. 10.1002/dvdy.20640 16317723

[B22] Lee J. E. Wang C. Xu S. Cho Y. W. Wang L. Feng X. (2013). H3k4 mono- and di-methyltransferase mll4 is required for enhancer activation during cell differentiation. eLife 2, e01503. 10.7554/eLife.01503 24368734 PMC3869375

[B23] Lefer A. M. Crossley K. Grigonis G. Lefer D. J. (1980). Mechanism of the beneficial effect of dexamethasone on myocardial cell integrity in acure myocardial ischemia. Basic Res. Cardiol. 75 (2), 328–339. 10.1007/BF01907581 7396811

[B24] Lellis-Santos C. Sakamoto L. H. Bromati C. R. Nogueira T. C. Leite A. R. Yamanaka T. S. (2012). The regulation of rasd1 expression by glucocorticoids and prolactin controls peripartum maternal insulin secretion. Endocrinology 153 (8), 3668–3678. 10.1210/en.2012-1135 22700767

[B25] Li P. Ge J. Li H. (2020). Lysine acetyltransferases and lysine deacetylases as targets for cardiovascular disease. Nat. Rev. Cardiol. 17 (2), 96–115. 10.1038/s41569-019-0235-9 31350538

[B26] Li Y. He L. Huang X. Bhaloo S. I. Zhao H. Zhang S. (2018). Genetic lineage tracing of nonmyocyte population by dual recombinases. Circulation 138 (8), 793–805. 10.1161/CIRCULATIONAHA.118.034250 29700121

[B27] Libby P. Maroko P. R. Bloor C. M. Sobel B. E. Braunwald E. (1973). Reduction of experimental myocardial infarct size by corticosteroid administration. J. Clin. Invest.. 52 (3), 599–607. 10.1172/JCI107221 4685084 PMC302298

[B28] Maddali M. Waje N. Arora N. Panchatcharam S. (2019). Effect of low-dose dexamethasone on extra vascular lung water in patients following on-pump elective primary coronary artery bypass graft surgery. Ann. Card. Anaesth. 22 (4), 372–378. 10.4103/aca.ACA_122_18 31621671 PMC6813707

[B29] McGrath M. F. Ogawa T. de Bold A. J. (2012). Ras dexamethasone-induced protein 1 is a modulator of hormone secretion in the volume overloaded heart. Am. J. Physiol. Heart Circ. Physiol. 302 (9), H1826–H1837. 10.1152/ajpheart.01085.2011 22408026

[B30] Narayanan N. Yang C. Xu A. (2004). Dexamethasone treatment improves sarcoplasmic reticulum function and contractile performance in aged myocardium. Mol. Cell. Biochem. 266 (1-2), 31–36. 10.1023/b:mcbi.0000049130.58074.73 15646025

[B31] Ng M. K. Celermajer D. S. (2004). Glucocorticoid treatment and cardiovascular disease. Heart 90 (8), 829–830. 10.1136/hrt.2003.031492 15253942 PMC1768346

[B32] Nojima M. Maruyama R. Yasui H. Suzuki H. Maruyama Y. Tarasawa I. (2009). Genomic screening for genes silenced by dna methylation revealed an association between rasd1 inactivation and dexamethasone resistance in multiple myeloma. Clin. Cancer Res. 15 (13), 4356–4364. 10.1158/1078-0432.CCR-08-3336 19549772

[B33] Pesant M. Sueur S. Dutartre P. Tallandier M. Grimaldi P. A. Rochette L. (2006). Peroxisome proliferator-activated receptor delta (ppardelta) activation protects h9c2 cardiomyoblasts from oxidative stress-induced apoptosis. Cardiovasc. Res. 69 (2), 440–449. 10.1016/j.cardiores.2005.10.019 16337160

[B34] Reeve J. L. Szegezdi E. Logue S. E. Ni C. T. O'Brien T. Ritter T. (2007). Distinct mechanisms of cardiomyocyte apoptosis induced by doxorubicin and hypoxia converge on mitochondria and are inhibited by bcl-xl. J. Cell. Mol. Med. 11 (3), 509–520. 10.1111/j.1582-4934.2007.00042.x 17635642 PMC3922357

[B35] Ross E. J. Linch D. C. (1982). Cushing's syndrome--killing disease: Discriminatory value of signs and symptoms aiding early diagnosis. Lancet 2 (8299), 646–649. 10.1016/s0140-6736(82)92749-0 6125785

[B36] Serrano M. Demarest B. L. Tone-Pah-Hote T. Tristani-Firouzi M. Yost H. J. (2019). Inhibition of notch signaling rescues cardiovascular development in kabuki syndrome. PLoS Biol. 17 (9), e3000087. 10.1371/journal.pbio.3000087 31479440 PMC6743796

[B37] Tsai H. J. Chiu C. H. Wang C. L. Chou C. H. (2010). A time-course study of gene responses of chicken granulosa cells to salmonella enteritidis infection. Vet. Microbiol. 144 (3-4), 325–333. 10.1016/j.vetmic.2010.01.004 20138717

[B38] Van Laarhoven P. M. Neitzel L. R. Quintana A. M. Geiger E. A. Zackai E. H. Clouthier D. E. (2015). Kabuki syndrome genes kmt2d and kdm6a: Functional analyses demonstrate critical roles in craniofacial, heart and brain development. Hum. Mol. Genet. 24 (15), 4443–4453. 10.1093/hmg/ddv180 25972376 PMC4492403

[B39] Wang K. Li Y. Qiang T. Chen J. Wang X. (2021). Role of epigenetic regulation in myocardial ischemia/reperfusion injury. Pharmacol. Res. 170, 105743. 10.1016/j.phrs.2021.105743 34182132

[B40] Wang L. H. Aberin M. Wu S. Wang S. P. (2021). The mll3/4 h3k4 methyltransferase complex in establishing an active enhancer landscape. Biochem. Soc. Trans. 49 (3), 1041–1054. 10.1042/BST20191164 34156443 PMC8286814

[B41] Wu B. Zhou B. Wang Y. Cheng H. L. Hang C. T. Pu W. T. (2010). Inducible cardiomyocyte-specific gene disruption directed by the rat tnnt2 promoter in the mouse. Genesis 48 (1), 63–72. 10.1002/dvg.20573 20014345 PMC2806493

[B42] Xu B. Strom J. Chen Q. M. (2011). Dexamethasone induces transcriptional activation of bcl-xl gene and inhibits cardiac injury by myocardial ischemia. Eur. J. Pharmacol. 668 (1-2), 194–200. 10.1016/j.ejphar.2011.06.019 21723861 PMC4148007

[B43] Xue Q. Patterson A. J. Xiao D. Zhang L. (2014). Glucocorticoid modulates angiotensin ii receptor expression patterns and protects the heart from ischemia and reperfusion injury. PLoS One 9 (9), e106827. 10.1371/journal.pone.0106827 25265380 PMC4180065

[B44] Yang G. Weng X. Zhao Y. Zhang X. Hu Y. Dai X. (2017). The histone h3k9 methyltransferase suv39h links sirt1 repression to myocardial infarction. Nat. Commun. 8, 14941. 10.1038/ncomms14941 28361889 PMC5381011

[B45] Yang L. Jin M. Jung N. Jeong K. W. (2020). MLL2 regulates glucocorticoid receptor-mediated transcription of ENACα in human retinal pigment epithelial cells.. Biochem. Biophys. Res. Commun. 525 (3), 675–680. 10.1016/j.bbrc.2020.02.046 32139118

[B46] Zhang L. Wang H. Zhao Y. Wang J. Dubielecka P. M. Zhuang S. (2018). Myocyte-specific overexpressing hdac4 promotes myocardial ischemia/reperfusion injury. Mol. Med. 24 (1), 37. 10.1186/s10020-018-0037-2 30134825 PMC6050730

[B47] Zheng Y. Huang Y. Mencius J. Li Y. Zhao L. Luo W. (2021). Distinct kinetic mechanisms of h3k4 methylation catalyzed by mll3 and mll4 core complexes. J. Biol. Chem. 296, 100635. 10.1016/j.jbc.2021.100635 33823156 PMC8144669

