# Supplementary material for: Histone methyltransferase KMT2D contributes to the protection of myocardial ischemic injury
Source: Front Cell Dev Biol. 2022 Jul 22;10:946484. doi: 10.3389/fcell.2022.946484 (PMC9354747; doi:10.3389/fcell.2022.946484)
Supplement: Supplementary file 1 [file DataSheet1.ZIP › Supplementary Materials/Supplementary Materials.docx]

***Supplementary Materials***

**Supplementary** **Figures and Tables**

**Figure S1**


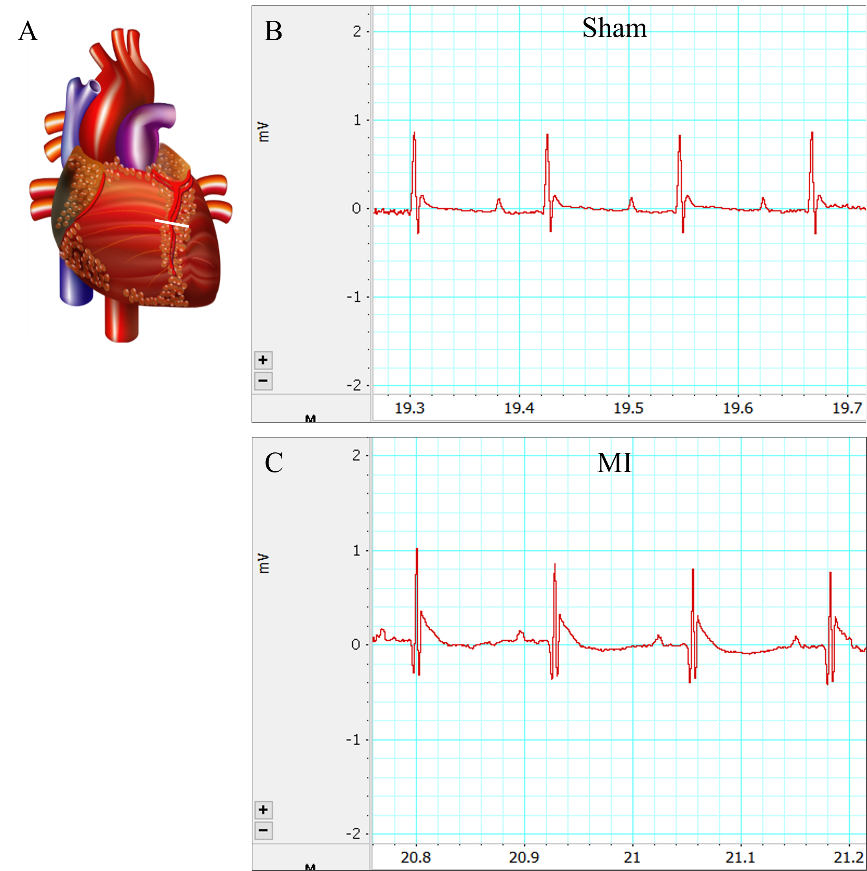


**Figure S1.** (A) Schematic diagram of ligation of left anterior descending coronary artery for artificial myocardial ischemia (MI) mice model. (B, C) Electrocardiogram of sham operated surgery (B) or MI-operated surgery (C).

**Figure S2**


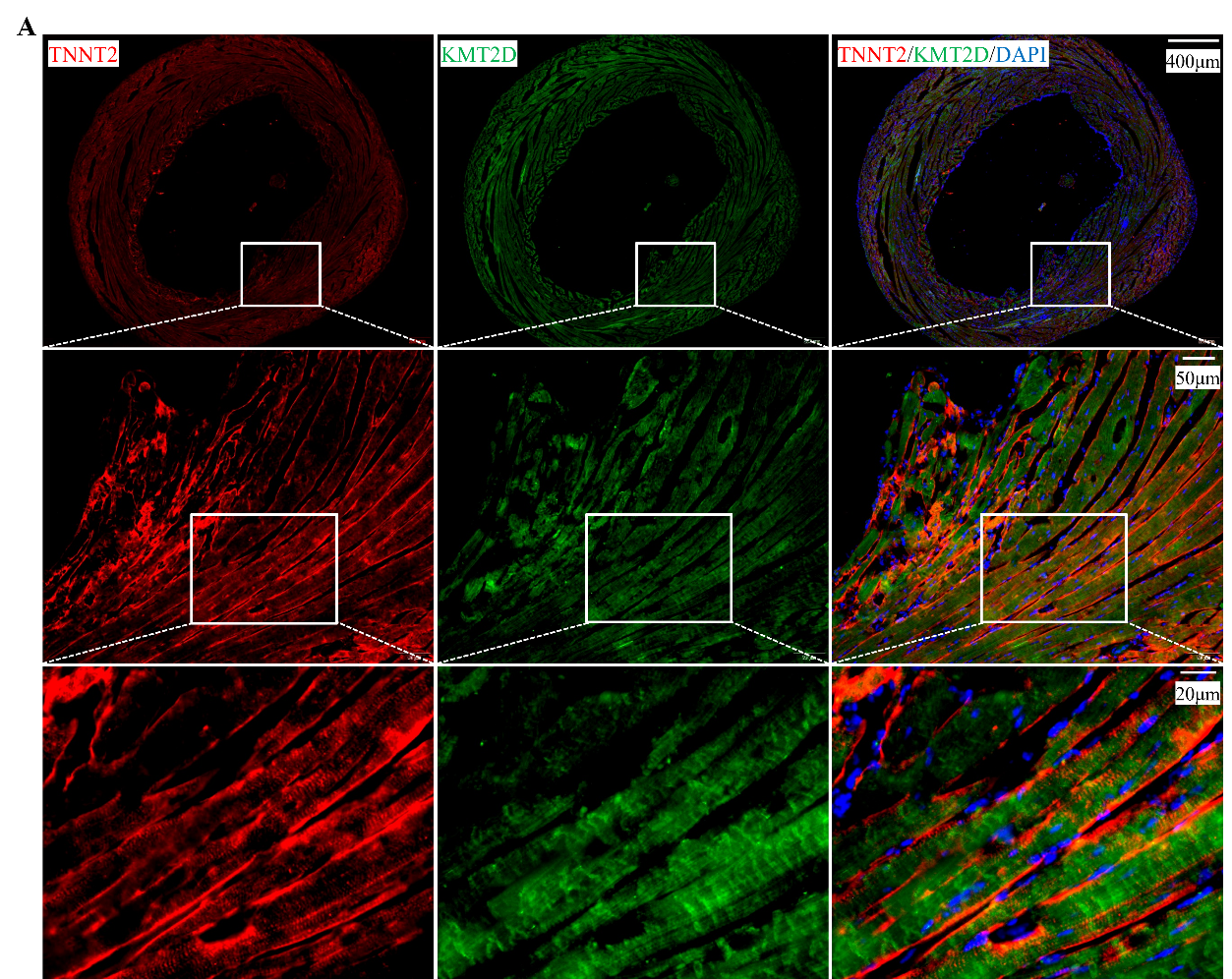

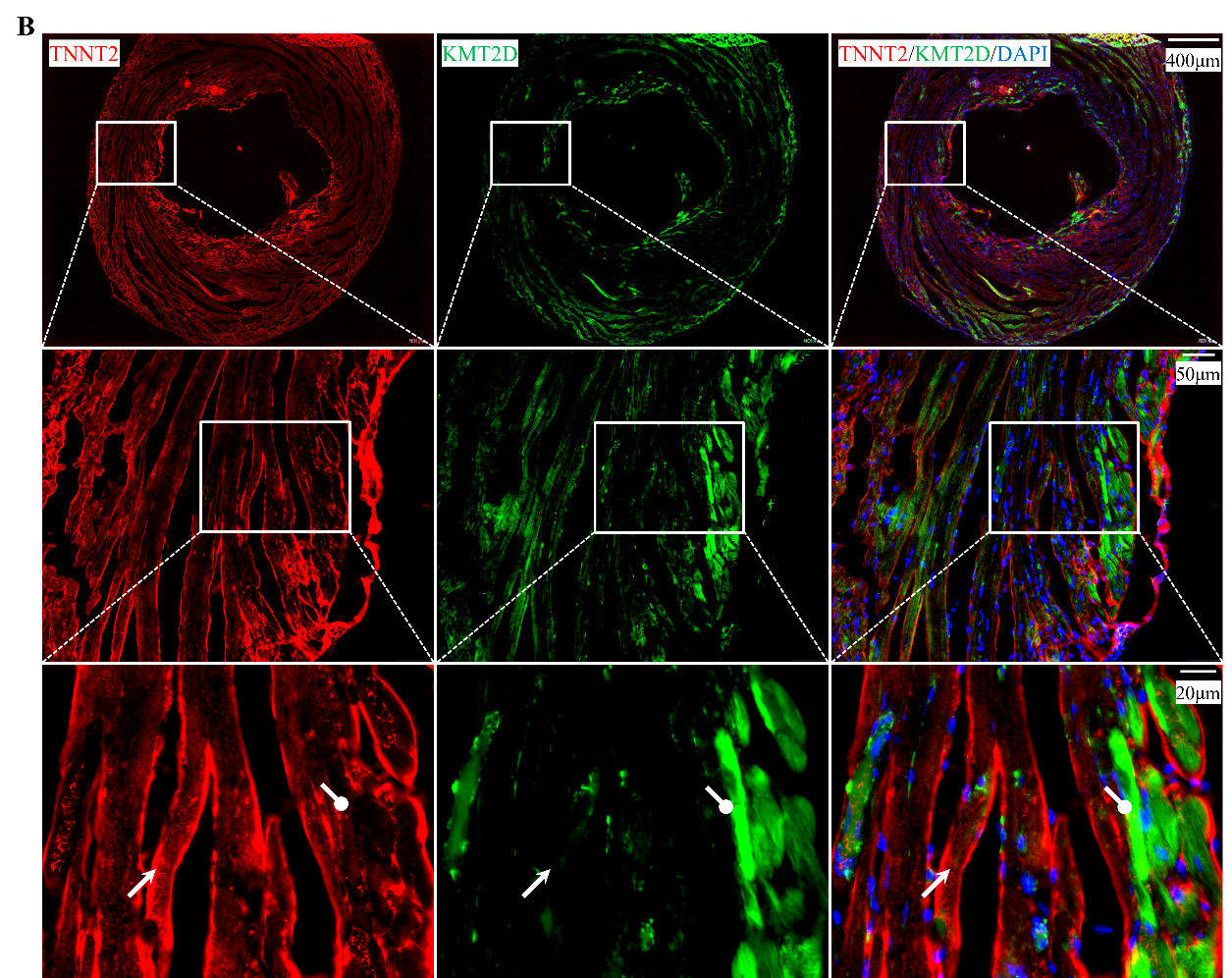


**Figure S2.** (A, B) Immunofluorescent double staining of KMT2D-Ctl (A) and KMT2D-cKO (B) heart tissues with antibodies to KMT2D and TNNT2. Red TNNT2, green KMT2D, and blue DAPI-stained cellular nuclei.

**Table S1.** Primers for genotyping.

| **Primers** | **Sequences** |
| --- | --- |
| *Kmt2d^flox/flox^* Forward: | 5’ ATTGCATCAGGCAAATCAGC 3’ |
| *Kmt2d^flox/flox^* Reverse: | 5’ GCAGAAGCCTGCTATGTCCA 3’ |
| *Tnnt2*-rtTA-Cre Forward: | 5’ CCGACCGATGTCAGCCTG 3’ |
| *Tnnt2*-rtTA-Cre Reverse: | 5’ CCCACCGTACTCGTCAATTCCAA 3’ |

**Table S2.** Primers for qPCR.

| **Primers** | **Sequences** |
| --- | --- |
| Mus *β-Actin* Forward: | 5’ GGTACCACCATGTACCCAGG 3’ |
| Mus *β-Actin* Reverse: | 5’ AGGGTGTAAAACGCAGCTCA 3’ |
| Rat *β-Actin* Forward: | 5’ CGCGAGTACAACCTTCTTGC 3’ |
| Rat *β-Actin* Reverse: | 5’ CCTTCTGACCCATACCCACC 3’ |
| Mus&Rat *Kmt2d* Forward: | 5’ GCGTTGTGTGGAGTGTATCG 3’ |
| Mus&Rat *Kmt2d* Reverse: | 5’ CACACCACTTGCACTTCCAG 3’ |
| Mus *Rasd1* Forward: | 5’ GCCGTTTCGAGGATGCTTAC 3’ |
| Mus *Rasd1* Reverse: | 5’ TCGCGGTTGTCTAAGCTGAA 3’ |
| Rat *Rasd1* Forward: | 5’ CATCCAAAGTGGGCAAGACG 3’ |
| Rat *Rasd1* Reverse: | 5’ GAAGGAGTCGCGGTTGTCTA 3’ |

**Table S3.** Primers of *Rasd1* GRE for ChIP-qPCR.

| **Primers** | **Sequences** |
| --- | --- |
| Mus *Rasd1* GRE Forward: | 5’ TCAGGTACATACTGTTCCCA 3’ |
| Mus *Rasd1* GRE Reverse: | 5’ TTCATACTCTCACAGCTTGC 3’ |
