# Supplementary figures and images for: Histone methyltransferase KMT2D contributes to the protection of myocardial ischemic injury
Source: Front Cell Dev Biol. 2022 Jul 22;10:946484. doi: 10.3389/fcell.2022.946484 (PMC9354747; doi:10.3389/fcell.2022.946484)

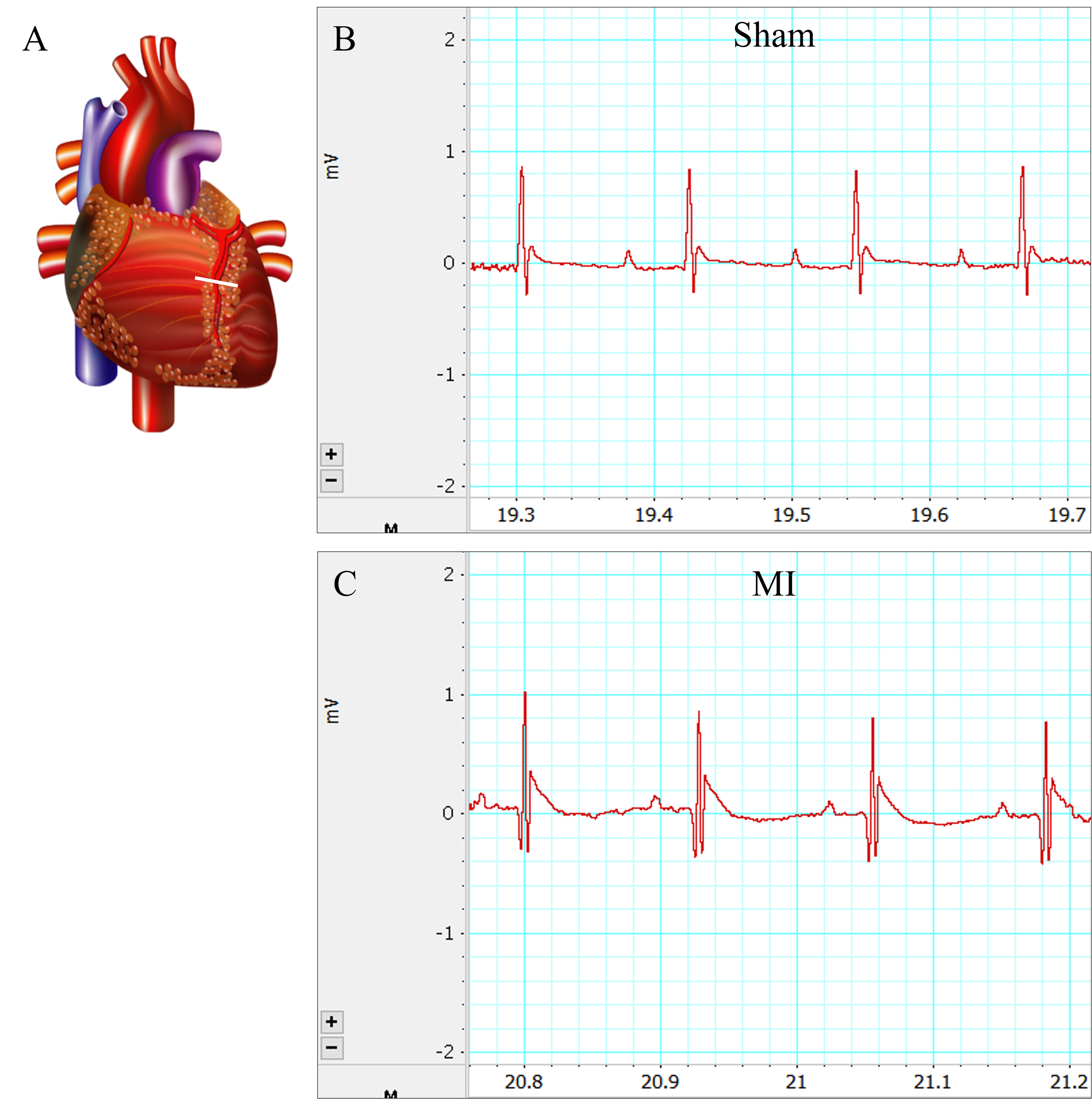

Supplement: Supplementary file 1 [file DataSheet1.ZIP › Supplementary Materials/Figure S1.tiff]

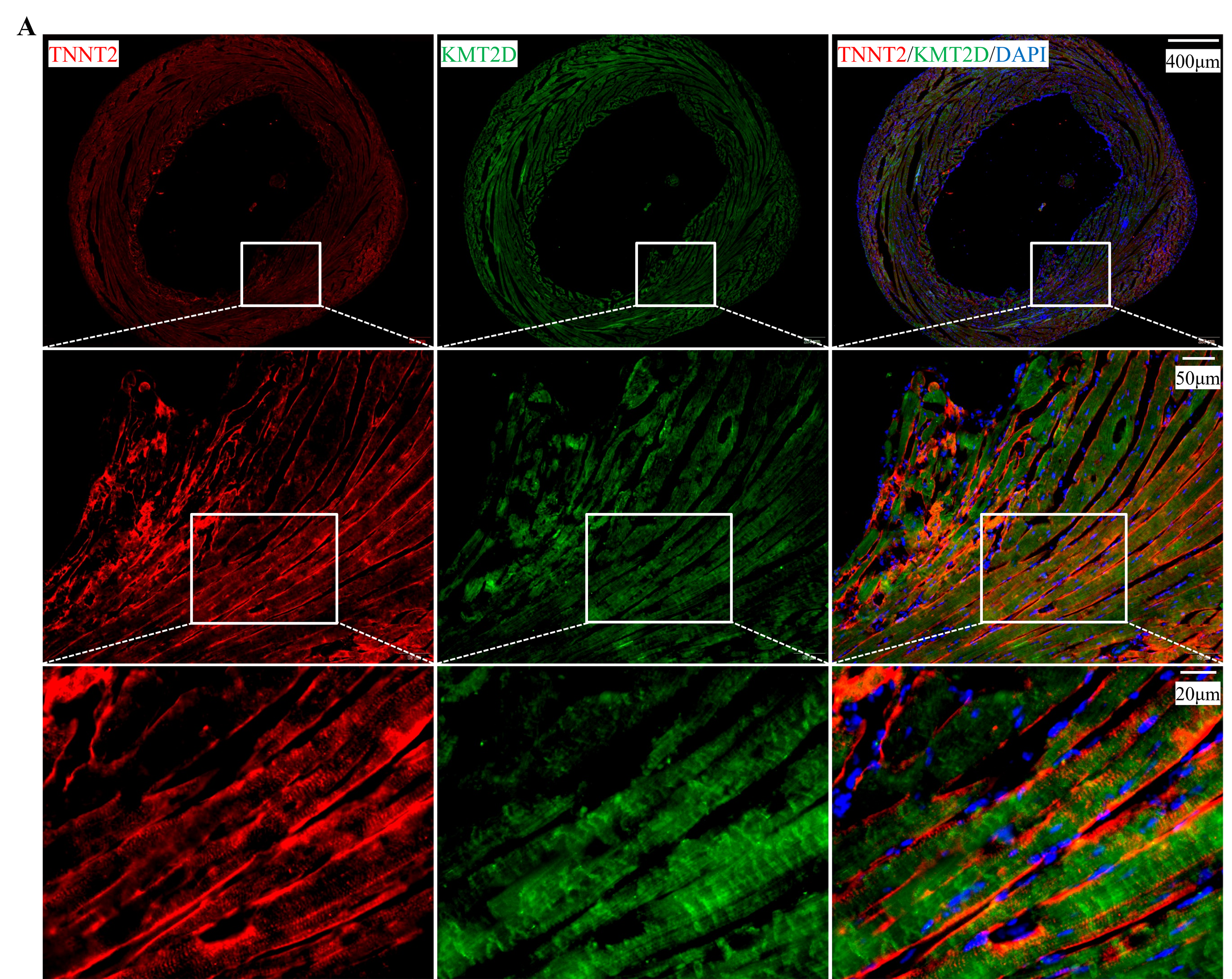

Supplement: Supplementary file 1 [file DataSheet1.ZIP › Supplementary Materials/Figure S2A.tif]

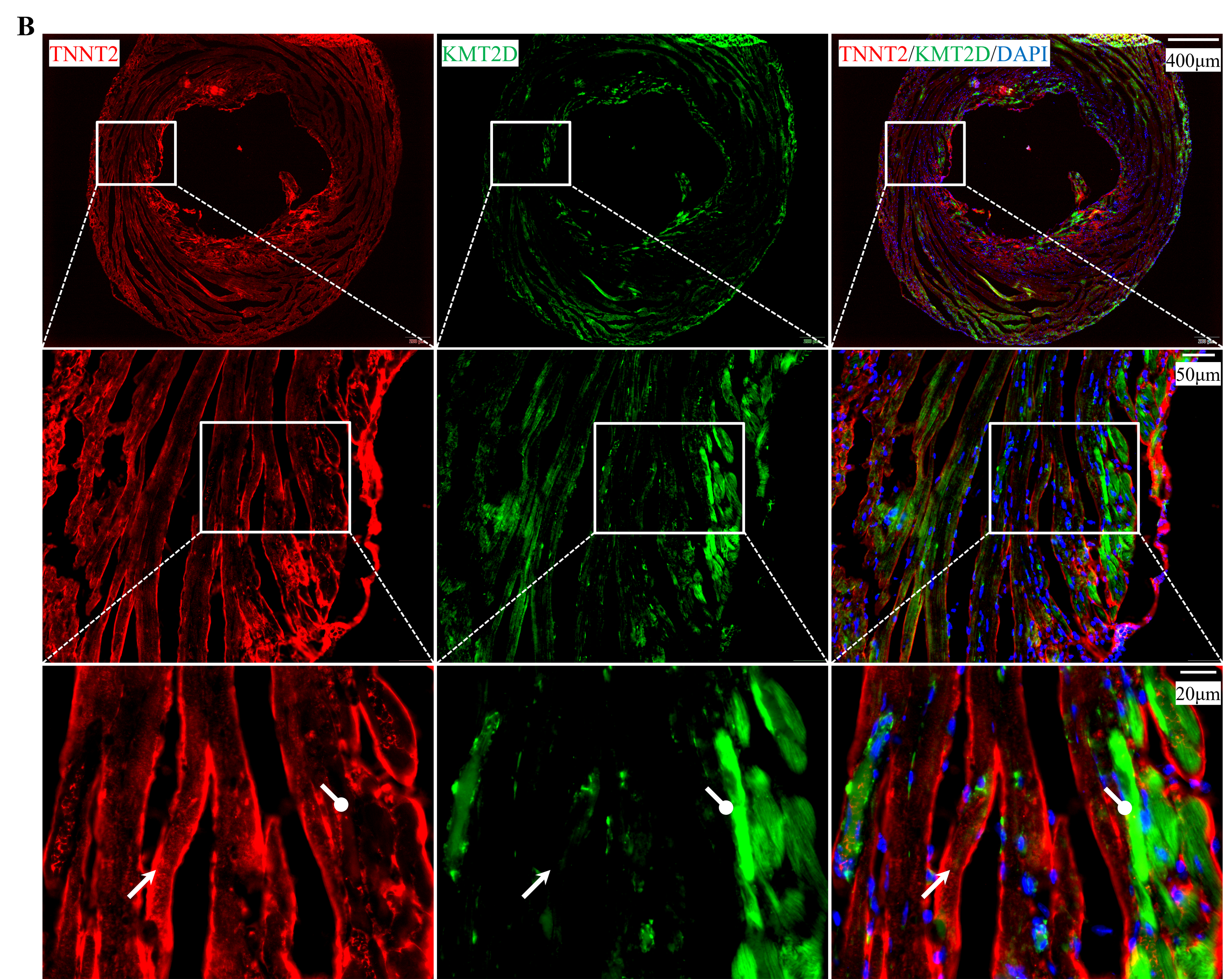

Supplement: Supplementary file 1 [file DataSheet1.ZIP › Supplementary Materials/Figure S2B.tiff]
